# *HRAS* is silenced by two neighboring G-quadruplexes and activated by MAZ, a zinc-finger transcription factor with DNA unfolding property

**DOI:** 10.1093/nar/gku574

**Published:** 2014-07-09

**Authors:** Susanna Cogoi, Andrey E. Shchekotikhin, Luigi E. Xodo

**Affiliations:** 1 Department of Medical and Biological Sciences, School of Medicine, P.le Kolbe 4, 33100 Udine, Italy; 2 Gause Institute of New Antibiotics, Russian Academy of Medical Sciences, B. Pirogovskaya, 11, Moscow 119021, Russia; 3 Mendeleyev University of Chemical Technology, 9 Miusskaya Square, Moscow 125190, Russia

## Abstract

The *HRAS* promoter contains immediately upstream of the transcription start site two neighboring G-elements, each capable of folding into a G-quadruplex structure. We have previously found that these G-quadruplexes bind to the zinc-finger transcription factors MAZ and Sp1. In the present study we have examined the interaction between the *HRAS* promoter and MAZ, demonstrating for the first time that the protein unfolds the G-quadruplex structures. We also demonstrate that MAZ-GST, in the presence of the complementary strands, promotes a rapid transformation of the two *HRAS* quadruplexes into duplexes. By a mutational analysis of the *HRAS* G-elements, we dissected the MAZ-binding sites from the quadruplex-forming motifs, finding that the two neighboring G-quadruplexes bring about a dramatic repression of transcription, in a synergistic manner. We also discovered that the two G-quadruplexes are strong targets for small anticancer molecules. We found that a cell-penetrating anthratiophenedione (ATPD-1), which binds tightly to the G-quadruplexes (Δ*T* > 15°C), promotes the total extinction of *HRAS* transcription. In contrast, when one of the two G-quadruplexes was abrogated by point mutations, ATPD-1 repressed transcription by only 50%. Our study provides relevant information for the rationale design of targeted therapy drugs specific for the *HRAS* oncogene.

## INTRODUCTION

The ras genes encode for GTP-binding proteins of 21 kDa (p21^RAS^) sharing a high degree of homology (1). Proteins p21^RAS^ regulate the response of the cell to a variety of extracellular stimuli including mitogens and differentiation factors (2). The ras genes have similar primary structures: five exons, the first of which is non-coding, conserved splicing sites and introns of different length and sequence (1). In many human tumors, the ras genes are transformed into oncogenes by point mutations, frequently occurring in exon 1, at codon 12, 13 or 61 (3). Mutated p21^RAS^ shows a decreased capacity to hydrolyze GTP to GDP, thus remaining locked into the activated state that constitutively stimulates cell proliferation (3). *HRAS* is frequently mutated in urinary bladder tumors (4) and its degree of overexpression correlates with tumor invasiveness (5,6). Mutations and overexpression contribute to the tumorigenesis of urinary bladder cancer (7). So far, the therapeutic strategies proposed to cure bladder cancer or to sensitize bladder cancer cells to conventional chemotherapy are based on the use of farnesyltransferase inhibitors. These compounds are able to block the binding of the ras protein to the cell membrane or inhibit the downstream RAS/MEK/ERK pathway, which stimulates cell growth in urinary cancer cells (8). In the present work we have focused on the promoter of the *HRAS* gene, in order to identify structure-function relationships that could be useful for the rationale design of anticancer drugs. We had previously found by chromatin immunoprecipitation that the transcription factors MAZ and Sp1 localize within the *HRAS* promoter, at two neighboring G-rich sequences named by us *hras*-1 and *hras*-2. Each sequence, being composed of runs of guanines (G-runs), folds into a stable G-quadruplex structure in the presence of potassium (9). Todd and Neidle (10) first discovered a correlation between quadruplex-forming sequences in the immediate upstream region of certain human genes and the occurrence of zinc-finger binding motifs (Sp1). After them, Kumar et al. (11) revealed that zinc-finger motifs (Sp1 and MAZ) co-localize with the quadruplex-forming sequences in human, chimpanzee, mouse and rat. In our laboratory we have shown that MAZ activates the expression of *HRAS* and *KRAS* in human and mouse (9,12). The function of MAZ in gene promoters is rather complex, as it can both activate (13,14) or inhibit (15–17) gene expression.

In this study we have investigated how MAZ and the *HRAS* quadruplexes influence transcription. We demonstrate for the first time that MAZ, a transcription factor that recognizes blocks of guanines, is able to unfold both the parallel and antiparallel *HRAS* quadruplexes and to promote their hybridization to the complementary C-rich strands, thus bringing back the duplex conformation: a critical step for the assembly of the transcription machinery. By a systematic mutational analysis of the *HRAS* promoter G-elements, we dissected the MAZ-binding sites from the quadruplex-forming motifs, finding that the two neighboring G-quadruplexes behave as a molecular on–off switch with a strong impact on transcription repression. We also report that the two quadruplex structures can function as targets for therapeutic molecules designed to repress oncogenic *HRAS* in bladder cancer cells. We have found that the *HRAS* promoter is completely blocked when both G-quadruplexes are targeted by an antrathiophenedione ligand (ATPD-1), whereas the promoter activity is reduced by ∼50% when only one G-quadruplex is targeted. We also provide a mechanistic insight on how ATPD-1 reduces *HRAS* gene expression. In summary, in this study we shed light on how *HRAS* transcription is regulated and how G4-DNA specific binders repress oncogenic *HRAS* in urinary bladder cancer cells.

## MATERIALS AND METHODS

### Oligonucleotides and fluorophore-labelled oligonucleotides

The following oligonucleotides, free and labeled at the 5′ and 3′ ends with FAM and TAMRA, have been purchased from Microsynth (CH), as HPLC purified compounds: 5′-TCGGGTTGCGGGCGCAGGGCACGGGCG (*hras*-1); 5′-CGGGGCGGGGCGGGGGCGGGGGCG (*hras*-2); 5′-CGCCCGTGCCCTGCGCCCGCAACCCGA (27Y); 5′-CGCCCCCGCCCCCGCCCCGCCCCG (23Y), 5′ F-TCGGGTTGCGGGCGCAGGGCACGGGCG-T (F-*hras*1-T); F-CGGGGCGGGGCGGGGGCGGGGGCG-T (F-*hras*2-T); where T = tetramethyl rhodamine; F = 6-carboxy-fluorescein. Oligonucleotide aliquots in milli-Q water were kept at -80°C.

### Recombinant MAZ-GST and EMSA

Recombinant MAZ tagged to glutathione S-tranferase (GST) was expressed in *Escherichia* *coli* BL21 DE3 plys by using plasmid pGEX-hMAZ (9). The bacteria were grown for 1–2 h at 37°C to an *A*_600_ of 0.6–0.8 prior to induction with isopropyl 1-thio-β-d-galactopyranoside (1 mM final concentration). Cells were allowed to grow with 4 μM Zn(CH_3_COO)_2_ for 18 h before harvesting. The cells were centrifuged at 6240 × *g*, 4°C, the supernatant was removed and the pellet was resuspended in phosphate buffered saline (PBS) containing 1 mM phenylmethylsulfonyl fluoride. The bacteria were lysed by sonication and centrifuged for 30′, 4°C, 34540 × *g*. Glutathione Sepharose 4B resin (GE Healthcare) (50% slurry in PBS) was added to the supernatant obtained in the previous step and incubated for 1 h, 4°C, on a shaker. The mix was centrifuged for 5 min at 500 × *g* and the pellet was washed 3× with PBS. MAZ-GST was eluted from the resin with a buffer composed by 50 mM Tris-HCl, pH 8 and 10 mM reduced glutathione. The purity and homogeneity were confirmed by sodium dodecyl sulphate-polyacrylamide gel electrophoresis (not shown). Protein concentration determined by Bradford method. The interaction between MAZ-GST and the *HRAS* duplexes in binding buffer (20 mM Tris-HCl pH 8, 8% glycerol, 100 μM MgCl_2_, 50 μM Zn-acetate, 1 mM DTT, 2.5 ng/μl polydIdC, 1 mM Na_3_VO_4_, 5 mM NaF; 1% phosphatase inhibitor cocktail, Sigma) added with KCl as specified in figure captions, was analyzed by electrophoretic mobility shift assays (EMSA), 5% polyacrylamide gel in Tris-borate (TB 1×) at 20°C, as previously described (12).

### DMS footprinting and circular dichroism

DMS footprinting of *hras*-1 and *hras*-2 in 100 mM CsCl and 140 mM KCl was performed as previously described (9). The CD spectra have been recorded on a JASCO J-600 spectropolarimeter equipped with a thermostatted cell holder, 3 μM oligonucleotide in 50 mM Tris-HCl, pH 7.4, 100 mM KCl.

### FRET experiments

FRET experiments with oligonucleotides F-*hras*1-T and F-*hras*2-T, tagged at the 5′ and 3′ ends with FAM and TAMRA, were carried out on a Microplate Spectrofluorometer System (Perkin Elmer 2300 Enspire, USA). Each sample contained 50 μl of 200 nM dual-labeled oligonucleotide in 50 mM Tris-HCl buffer, pH 7.4, 50 μM Zn acetate, KCl and MAZ-GST, as specified in the figure captions. The samples were incubated at 37°C as specified in the text. Emission spectra were obtained setting the excitation wavelength at 480 nm and recording the emission from 500 to 650 nm. FRET-melting experiments were performed on a real-time polymerase chain reaction apparatus (CFX96, BioRad, Hercules, CA). FRET-melting experiments were obtained as follows: 5 min equilibration at 20°C, 5 min; stepwise increase of temperature up to 95°C (1°C/min); number of cycles, 76.

FRET efficiency (*E*) was calculated from the fluorescence intensity of the donor D in the presence (*I*_DA_) and absence (*I*_D_) of the acceptor as:

\begin{equation*} E = 1 - \frac{{I_{{\rm DA}} }}{{I_{\rm D} }} \end{equation*}

*I*_DA_ and *I*_D_ were measured in same buffer under identical concentrations (*I*_D_ was obtained by transforming the dual-labeled oligonucleotide into the corresponding duplex in which the fluorophores are at a distance for which FRET = 0). FRET efficiency values were converted to distances between donor and acceptor by using:

\begin{equation*} R = R_0 \sqrt[6]{{\frac{1}{E} - 1}} \end{equation*}

where *R* is the distance (Å) and *R*_0_ is the Föster distance (defined as the distance at which energy transfer is 50% of the maximum value). The Föster distance (*R*_0_) between FAM and TAMRA is 50 Å (18–20).

### Filter binding assay

We performed a double-filter method as previously described (21). A nitrocellulose membrane was incubated for 10 min in 0.4 M KOH at 4°C, washed with water and equilibrated in 20 mM Tris HCl pH 8, 8% glycerol; Immobilon-NY+ (Millipore) was equilibrated in the same buffer. Immobilon membrane was placed below the nitrocellulose membrane in a filter binding apparatus Minifold I (Whatman) where the upper membrane captures MAZ-GST with or without bound DNA and the lower membrane traps free DNA. Samples containing DNA and MAZ-GST, incubated at 4 °C for 30 min in binding buffer (see EMSA) added with KCl as specified in figure captions, were applied to filters under vacuum and washed with binding buffer. Filters were dried, exposed for autoradiography and quantified using an Image Scanner controlled by ImageQuant TL V2003.03 software (GE Healthcare).

### Dual luciferase assays

Transfection was performed by mixing vector (250 ng/well) pHRAS-luc or mutant vectors Mut-1, Mut-2, Mut-A, Mut-B, Mut-C and Mut-D with 10 ng of control plasmid pRL-CMV where *Renilla* luciferase was driven by the CMV promoter (10 ng/well) using jet-PEI (Polyplus) as a transfecting reagent. Vectors pHRAS-luc and mutant vectors expressed firefly luciferase under the control of wild-type or mutant *HRAS* promoters (Figure 5 shows the mutations) . Firefly luciferase in cell lysates was measured and normalized for *Renilla* luciferase. Luciferase assays were performed 48 and 72 h after transfection with Dual-Glo Luciferase Assay System (Promega) following the supplier instructions. Samples were read on a Turner Luminometer and the relative luminescence expressed as (T/C × 100) where T = firefly luciferase/renilla luciferase in treated cells and C = firefly luciferase/renilla luciferase in untreated cells.

**Figure 1.**
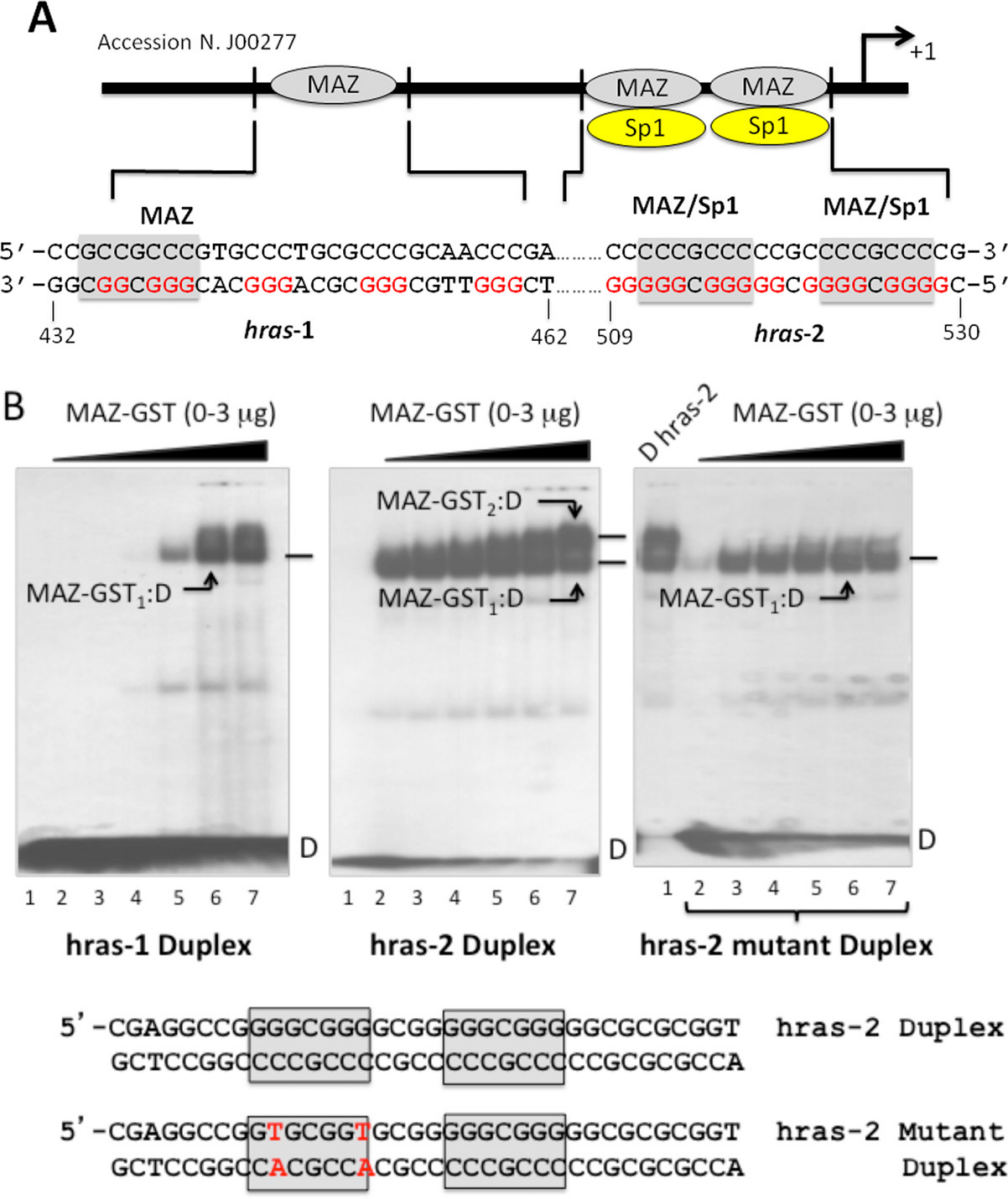
(**A**) Structure of the *HRAS* promoter upstream of TSS. The sequences of *hras*-1 (432–462, J00277) and *hras*-2 are shown. The binding sites of MAZ and Sp1 are evidenced; (**B**) EMSA showing the binding of MAZ-GST to *hras*-1 duplex, *hras*-2 duplex and *hras*-2 mutant duplex (see sequences in panel A) in binding buffer (‘Materials and Methods’ section). Left and middle gels were loaded as follows: 0, 0.5, 1, 1.5, 2, 2.5 and 3 μg MAZ-GST and duplex in lanes 1 to 7, respectively. Right gel was loaded as follows: 3 μg MAZ-GST and wild-type duplex (lane 1); 0.5, 1, 1.5, 2, 2.5 and 3 μg MAZ-GST + mutant *hras*-2 duplex in lanes 2 to 7, respectively.

**Figure 2.**
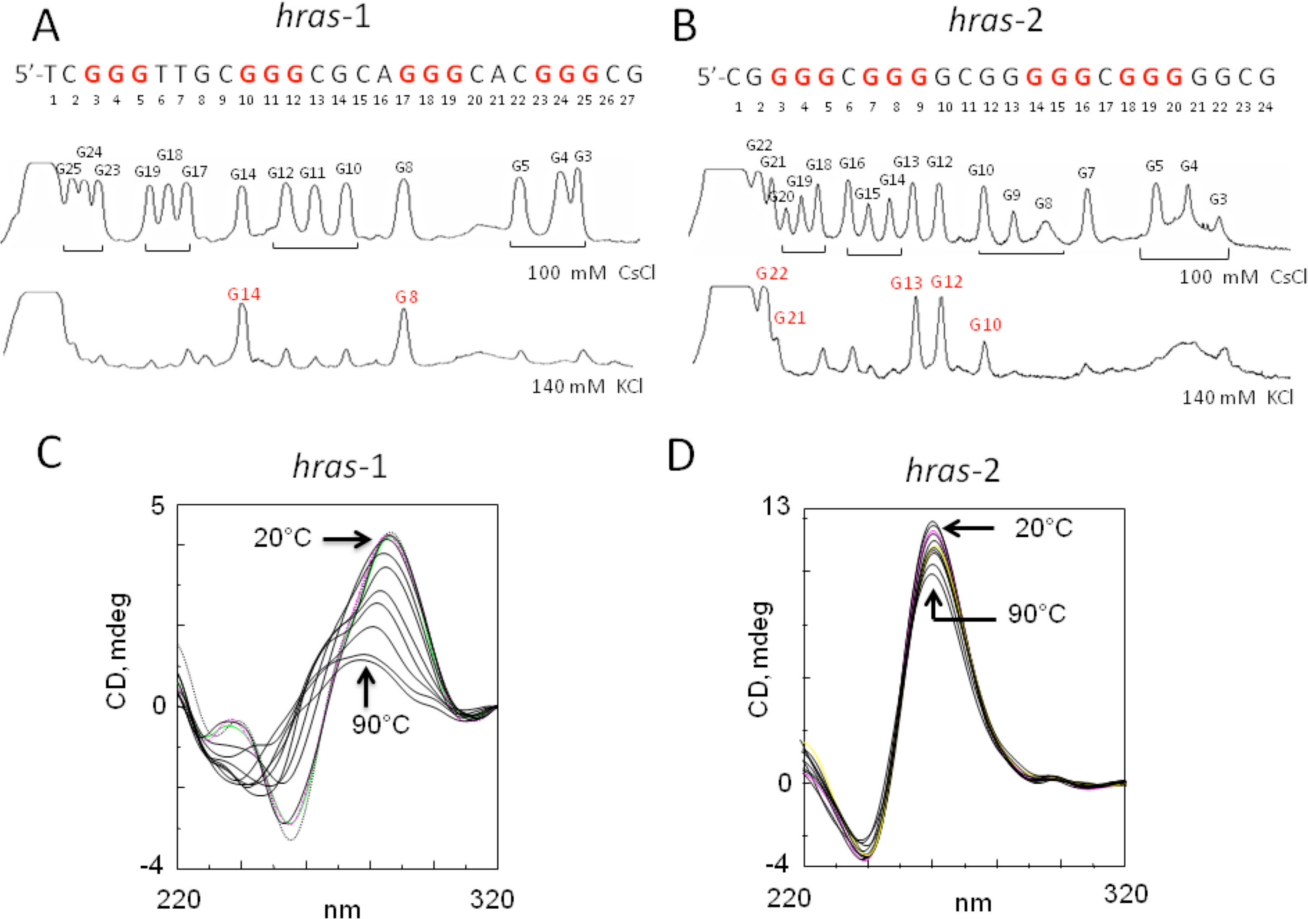
(**A**) Sequence of *hras*-1 and densitometric scans of DMS-footprintings in 100 mM CsCl and 140 mM KCl; (**B**) Sequence of *hras*-2 and densitometric scans of DMS-footprinting in 100 mM CsCl and 140 mM KCl; (**C**) CD spectra of *hras*-1 in 50 mM Tris-HCl pH 7.4, 100 mM KCl, T = 20, 30, 40, 50, 55, 60, 65, 70, 80 and 90°C; (**D**) CD spectra of *hras*-2 in 50 mM Tris-HCl pH 7.5, 100 mM KCl, *T* = 20, 30, 40, 50, 55, 60, 65, 70, 80 and 90°C.

**Figure 3.**
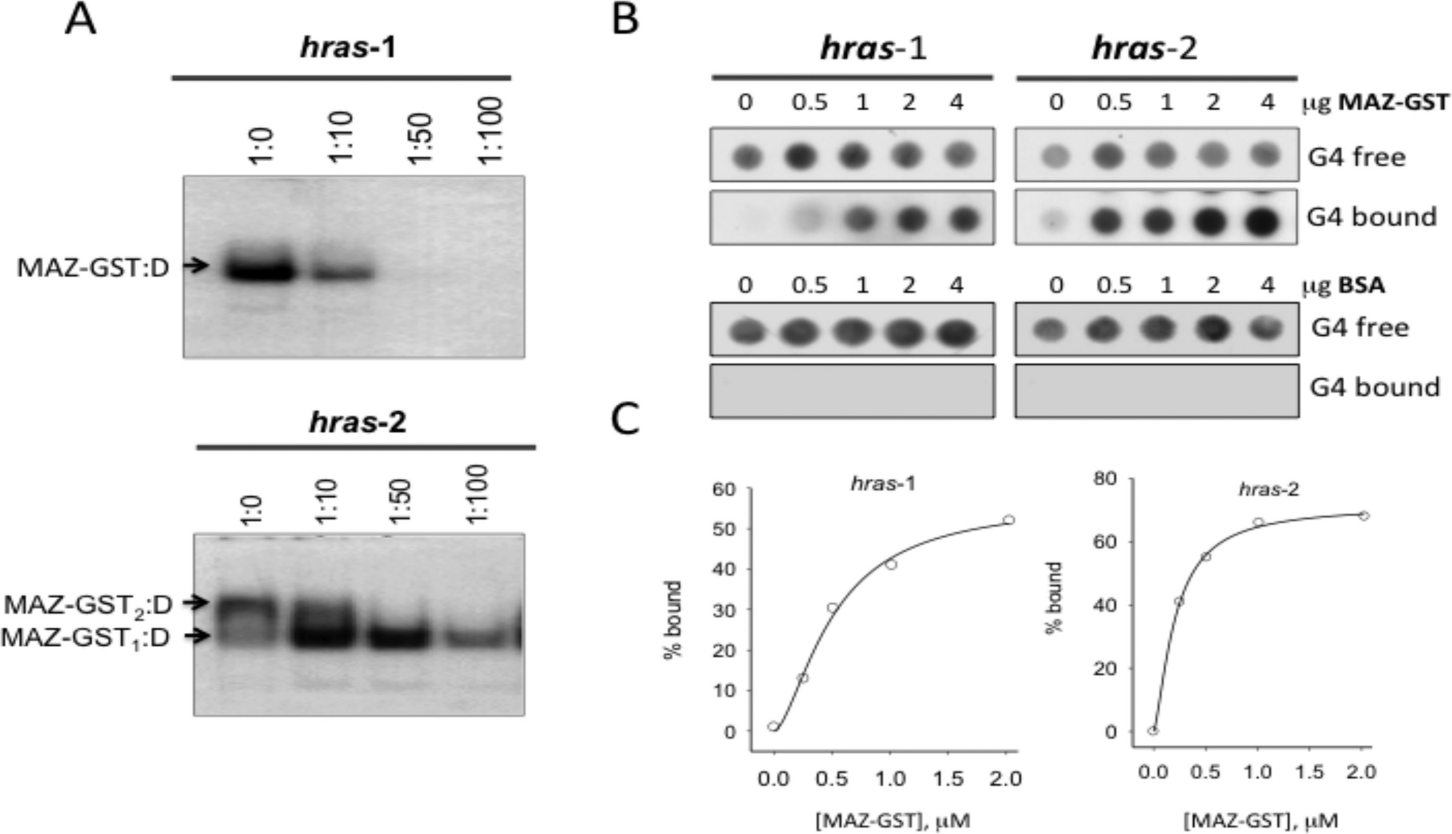
(**A**) EMSA competition assay. The DNA–protein complexes between radiolabeled duplex *hras*-1 and duplex *hras*-2 and MAZ-GST are competed away by excess (10-, 50- and 100-fold) cold G-quadruplex; (**B**) Typical dot-blot assay showing the binding of MAZ-GST to quadruplex *hras*-1 and *hras*-2 in binding buffer added with 100 mM KCl. Free and bound G-quadruplexes, immobilized in nylon and nitrocellulose respectively, are shown at various MAZ-GST concentrations. As a control a dot blot with BSA was performed. (**C**) The % of quadruplex bound to MAZ-GST is plotted against the protein concentration. The experimental points (average of two experiments, uncertainty ± 15%) have been best-fitted to the Hill equation {f = [L]^n^/([L]^n^+ K_D_)} (Sigma Plot 11).

**Figure 4.**
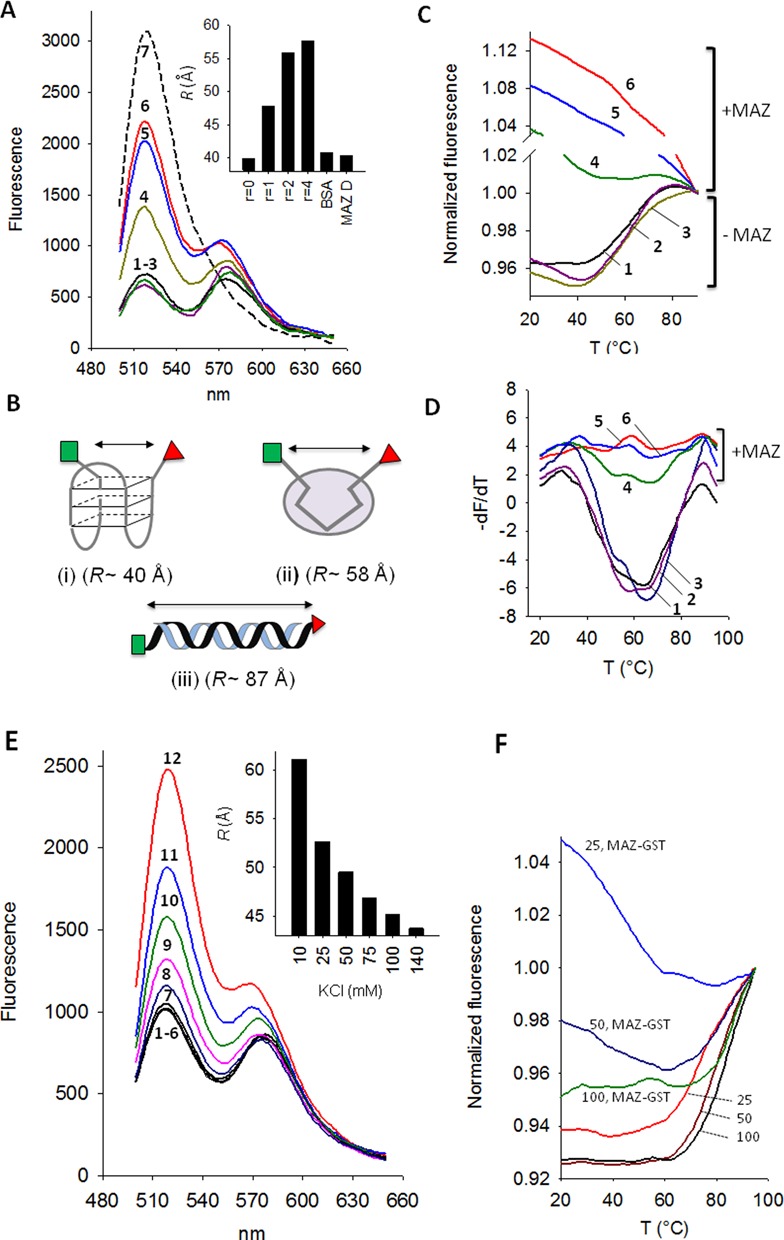
(**A**) Fluorescence emission of 200 nM quadruplex *hras*-1 tagged at the 5′ and 3′ ends with FAM and TAMRA in 50 mM Tris-HCl, pH 7.4, 50 mM KCl, Ex = 480 nm. Spectra: **1**, F-*hras*1-T; **2**, F-*hras*1-T + heated MAZ-GST (1:4); **3**, F-*hras*1-T + BSA (1:4); **4–6**, F-*hras*1-T + MAZ-GST at *r* = 1, 2 and 4, respectively; **7**, F-*hras*1-T hybridized to complementary 27Y strand to give the duplex. Each quadruplex–protein mixture has been incubated for 2 h before FRET analysis. The inset shows the end-to-end distance (*R*, Å) of quadruplex *hras*-1 treated for 2 h with MAZ-GST (*r* = 0, 1, 2 and 4), with BSA (*r* = 4) and with denatured MAZ-GST (*r* = 4) (MAZ D); (**B**) cartoon showing the three forms of *hras*-1 and for each the calculated end-to-end distance; (**C**) Normalized fluorescence *versus* T curves of quadruplex *hras*-1 in 50 mM Tris-HCl pH 7.4, 50 mM KCl: curve **1**, quadruplex *hras*-1; curve **2**, quadruplex *hras*-1 treated for 2 h with heated MAZ-GST (*r* = 4); curve **3**, quadruplex *hras*-1 treated for 2 h with BSA (*r* = 4); curves **4**, **5** and **6**, quadruplex *hras*-1 treated for 2 h with MAZ-GST at *r* = 1, 2 and 4, respectively; (**D**) –dF/dT *versus* T curves as in C; (**E**) FRET of *hras*-2 tagged with FAM and TAMRA (F-*hras*2-T) in 10, 25, 50, 75, 100, 140 mM KCl (curves **1–6**) and after treatment with MAZ-GST (*r* = 4) (curves **12** to **7**). The inset shows the decrease of the end-to-end distance of quadruplex *hras*-2 treated with MAZ-GST (*r* = 4) at various KCl concentrations; (**F**) Normalized fluorescence *versus* T curves of quadruplex *hras*-2 in 25, 50 and 100 mM KCl with and without treatment with MAZ-GST (*r* = 4) for 2 h.

**Figure 5.**
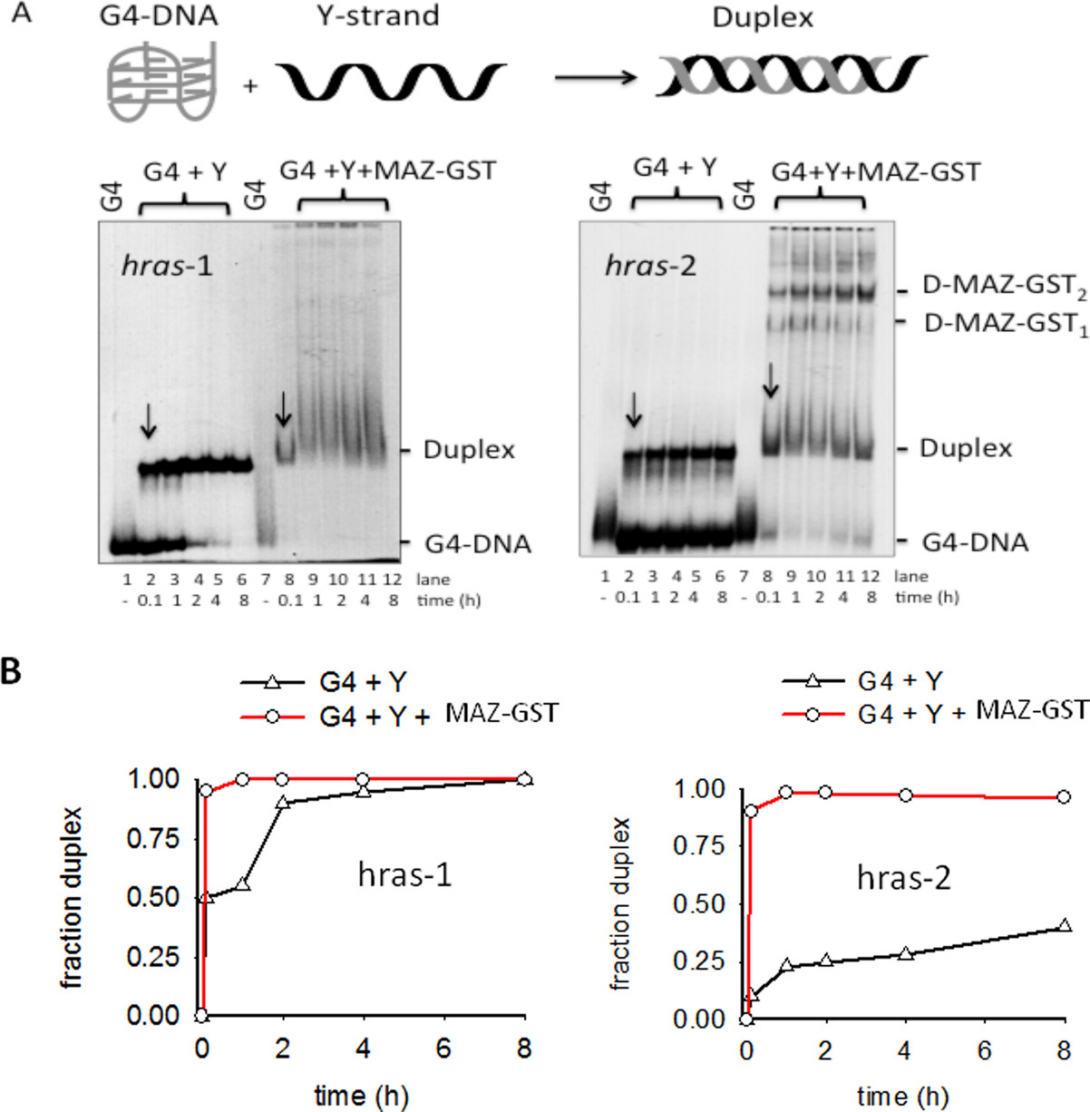
(**A**, left) Annealing between radiolabeled quadruplex *hras*-1 and complementary Y-strand in binding buffer (‘Materials and Methods’ section) added with 100 mM KCl. The amount of MAZ-GST was 3 μg; (A, right) Annealing between radiolabeled quadruplex *hras*-2 and complementary Y-strand in binding buffer added with 30 mM KCl. The amount of MAZ-GST was 3 μg. EMSA has been carried out with 12% PAGE in TB at 20°C; (**B**) Plots showing the fraction of duplex formed in the presence or absence of MAZ-GST, as a function of time.

Co-transfection experiments in T24 cells were carried out by using mixtures containing 100 ng of pHRAS-luc or mutant vector, 10 ng of pRL-CMV and 100 ng of pCMV-MAZ or pcDNA3 plasmid (empty vector) as mass for control transfections. Each transfection was performed in triplicate. Plasmid pHRAS-luc, Mut-1 and Mut-2 were previously constructed in our laboratory (9). Instead, Mut-A, Mut-B, Mut-C and Mut-D, obtained from pHRAS-luc by site-directed mutagenesis, have been constructed by GenScript, Piscataway, NJ.

## RESULTS AND DISCUSSION

The *HRAS* promoter, upstream of the transcription start site (TSS), contains several copies of the G-box 5′-GGGCGGG, which is recognized by the transcription factors MAZ and Sp1 [the consensus for MAZ is 5′-(G/C)GG(C/A)GGG, while the consensus for Sp1 is 5′-GGGCGGG (22–24)]. Upstream of the transcription start sites there are two G-elements, *hras*-1 and *hras*-2, the first of which contains one mismatched G-box, 5′-GGGCGGC, and the second two perfect G-boxes, 5′-GGGCGG (Figure 1A). We have previously demonstrated by chromatin immunoprecipitation that under *in vivo* conditions MAZ is associated with both *hras*-1 and *hras*-2 sequences but Sp1 only to *hras*-2 (9). This was also confirmed by biotin-streptavidin pull-down and western blot assays: compared to the amount of proteins in the nuclear extract, duplex *hras*-2 enriched the eluted fractions of both MAZ and Sp1, whereas duplex *hras*-1 only enriched MAZ (Supporting Information S_1_). Together, these experiments identified MAZ as a specific interactor of both *hras*-1 and *hras*-2 sequences.

As MAZ recognizes both *HRAS* G-elements, in the present study we focus mainly on this zinc-finger transcription factor. The binding of MAZ to duplexes *hras*-1 and *hras*-2 was examined by EMSA, using recombinant MAZ-GST (Figure 1B). It can be seen that 0.5 μg of MAZ-GST is sufficient to detect its binding to duplex *hras*-2, while the binding to duplex *hras*-1 is observed at 2 μg protein. This suggests that MAZ-GST has more affinity for *hras*-2 than for *hras*-1 (see also S_1_). Moreover, in agreement with the fact that it harbors two MAZ-binding sites, duplex *hras*-2 forms two DNA–protein complexes, one with a 1:1 stoichiometry, the other with a 1:2 stoichiometry. As we expected, when one of the two MAZ-binding sites was abrogated by point mutations, the 1:2 complex was not detected in the gel (Figure 1B).

As sequences *hras*-1 and *hras*-2 are composed of runs of guanines, they are inclined to fold into G-quadruplex structures (Figure 2A–D) (9). DMS-footprinting was used to determine that these guanines are involved in the formation of G-quadruplexes. All *hras*-1 guanines are reactive to DMS in 100 mM CsCl, while in 140 mM KCl only G8 and G14 are reactive. This unambiguously shows that G3–G5, G10–G12, G17–G19, G23–G25 are involved in the formation of the G-tetrad core of the quadruplex. *Hras*-1 shows a CD spectrum with positive and negative ellipticities at 295 and 256 nm, respectively, suggesting that the sequence folds into an antiparallel quadruplex. CD and FRET experiments as a function of temperature showed that *hras*-1 melts in a cooperative manner with a biphasic profile in 100 mM KCl, indicating that it folds into two quadruplexes with *T*_M_'s of ∼53 and ∼65°C. Moreover, due to its moderate stability, quadruplex *hras*-1 is unable to arrest Taq polymerase in primer extension experiments, but it does so in the presence of a G4 stabilizer (phthalocyanine) (S_2_,a).

Sequence *hras*-2, being composed of guanine blocks of 4 and 5 bases, can also fold into a G-quadruplex (Figure 2B,D). In CsCl all guanines react with DMS, but in 140 mM KCl the reactivity is only observed with G10, G12, G13, G21 and G22. This indicates that G3–G5, G7–G9, G14–G16 and G18–G20 should be engaged in the formation of the quadruplex. As the CD of *hras*-2 is dominated by a strong positive ellipticity at 260 nm, we concluded that it forms a parallel G-quadruplex. The *T*_M_ of this structure in 20 mM KCl is ∼77°C, but in 100 mM KCl is >90°C. Primer extension experiment showed that quadruplex *hras*-2 caused a strong stall of Taq polymerase, even in the absence of a G4 stabilizer, thanks to its high stability (S_2,b_).

Recent bio-informatic studies have shown that zinc-finger transcription factors, such as MAZ and Sp1, co-localize with quadruplex-forming motifs (11,25). In keeping with this, we have previously found by EMSA that recombinant MAZ-GST binds to both quadruplexes formed by *hras*-1 and *hras*-2 (9). Here, we examined the binding of MAZ-GST to quadruplexes *hras*-1 and *hras*-2 also by a competition binding assay. Figure 3A shows that the DNA-protein complexes between MAZ-GST and ^32^P-labeled *hras*-1 and *hras*-2 duplexes are competed away by 10, 50, 100-fold excess of cold (*hras*-1 or *hras*-2) G-quadruplex. As duplex *hras*-1 has only one MAZ-binding site, it forms with MAZ-GST a 1:1 complex which is competed by a 10-fold excess of cold quadruplex *hras*-1. Duplex *hras*-2, having two protein-binding sites, forms both 1:1 and 1:2 complexes with MAZ-GST. The latter complex is competed by a 10-fold excess cold quadruplex *hras*-2, suggesting that both *HRAS* quadruplexes compete to a similar extent with their duplex sequences. Instead, the 1:1 complex is competed by a 100-fold excess G-quadruplex. This is in agreement with the higher stability of the *hras*-2 duplex complex compared to the *hras*-1 duplex complex.

To estimate the affinity of MAZ-GST for quadruplexes *hras*-1 and *hras*-2, we have carried out a double-filter binding assay, as previously described (21). The G-quadruplexes end-labeled with ^32^P and MAZ-GST have been incubated in binding buffer containing 100 mM KCl (see ‘Materials and Methods’ section). Free and MAZ-GST-bound quadruplexes were separated and immobilized on nylon and nitrocellulose, respectively. Typical dot blots are shown in Figure 3B. The fractions of bound quadruplexes were plotted against MAZ-GST concentrations and the data best-fitted to the Hill equation (Figure 3C). For quadruplex *hras*-1 we obtained *K*_D_ = 504 ± 84 nM, *n* = 1.6 ± 0.3, while for quadruplex *hras*-2 we obtained *K*_D_ = 207 ± 12 nM and *n* = 1.4 ± 0.2, where *n* is the cooperative coefficient. As *n* > 1, the binding between MAZ-GST and the quadruplexes is slightly cooperative. As a control, we incubated the G-quadruplexes with bovine serum albumin (BSA) and no binding was detected in the nitrocellulose membrane, as expected. The *K*_D_ values measured in this way presumably underestimate the affinity of MAZ-GST for the two G-quadruplexes as: (i) recombinant bacterially expressed proteins do not carry the post-translational modifications of the cellular analogues; (ii) upon binding to the protein, the quadruplex is unfolded and assumes a conformation for which the MAZ shows a lower affinity (see S3). The affinity of MAZ-GST for the parallel *hras*-2 quadruplex is 2.4-fold higher than for the antiparallel *hras*-1 quadruplex. The *K*_D_'s between MAZ-GST and *HRAS* quadruplexes are not so different from those reported for the binding of recombinant UP1, hRNP A1, nucleophosmin and nucleolin to G4-DNA (26–28). Instead, the *K*_D_ reported for the interaction between cellular Sp1 and quadruplex cKIT is >10-fold lower (29).

As MAZ binds to both quadruplex and duplex conformations of *hras*-1 and *hras*-2, we wanted to find out for which one it shows more affinity. We found an answer in the biotin-streptavidin pull-down/western blot assays which was done with biotin-labeled *hras*-1, *hras*-2 and scrambled oligonucleotides as probes. Based on the ionic strength of the NaCl solutions used for separating the protein from the DNA probes, we concluded that the affinity of MAZ decreases as follows: *HRAS* duplex > *HRAS* quadruplex >> scrambled duplex or scrambled single strand (S_3_).

Next, we interrogated if recombinant MAZ-GST is able to unfold the *HRAS* G-quadruplexes. The unfolding process can be followed by fluorescence-resonance energy transfer (FRET), using *hras*-1 and *hras*-2 quadruplex-forming sequences tagged at the 5′ and 3′ ends with FAM (donor) and TAMRA (acceptor) [F-*hras*1-T, F-*hras*2-T] (26,30,31). In Figure 4A we report the data obtained with the antiparallel *hras*-1 quadruplex. In the presence of 50 mM KCl, by exciting the donor at 480 nm, both donor (520 nm) and acceptor (575 nm) emit fluorescence, thanks to the FRET effect (spectrum 1, quadruplex). The energy transfer efficiency (*E*) of the folded state (0.79) was used to measure the end-to-end distance in quadruplex *hras*-1, ∼40 Å [Figure 4B (i)]. This value is consistent with the dimensions of the G-quadruplex [diameter ∼25 Å, height 6.3 Å (32)], accounting for the presence at the quadruplex ends of two extra nucleotides and the dye linkers. When the G-quadruplex is hybridized to its complementary strand (27Y), it loses its folded conformation and assumes a linear B-DNA duplex form. A short B-DNA duplex behaves as rigid rod, its contour length can be calculated from the distance between two base pairs of 3.4 Å. In duplex *hras*-1 the donor and acceptor are separated by ∼87 Å, a distance at which FRET = 0 (spectrum 7, Figure 4A). To understand if quadruplex F-*hras*1-T is unfolded by MAZ-GST, we incubated for 2 h the quadruplex and protein at *r* = 1, 2 and 4 (*r* = [protein]/[quadruplex]) in 50 mM KCl and measured the fluorescence between 500 and 650 nm (spectra 4–6, Figure 4A). It can be seen that MAZ-GST brings about a dramatic increase of the donor emission, resulting from an increase of the reciprocal distance between donor and acceptor: a structural change consistent with the disruption of the G-quadruplex. The end-to-end distance (*R*) of quadruplex *hras*-1 increases with *r* from ∼40 up to ∼58 Å (inset). Comparing *R* obtained with *hras*-1 in the duplex conformation (∼87 Å) with *R* of *hras*-1 bound to MAZ-GST (58 Å, at *r* = 4), we may conclude that the quadruplex is open but not fully extended by the protein [Figure 4B (ii-iii)]. In other words, the G-quadruplex in the DNA–protein complex should be unfolded but with the 5′ and 3′ ends within the Föster distance. That is why some emission at 575 nm is still present in the samples treated with MAZ-GST. As a control, we heated MAZ-GST before being used and also replaced it with BSA (unspecific protein). In both cases no increase of donor fluorescence at 520 nm was observed (spectra 2 and 3). To further support our interpretation, we performed FRET-melting experiments, reasoning that an unfolded quadruplex would not give the typical quadruplex-to-single strand transition. Figure 4C,D shows the melting curves (*F* versus *T* and –d*F*/d*T* versus *T*) of quadruplex F-*hras*1-T in 50 mM KCl before (curve 1) and after incubation for 2 h with MAZ-GST at *r* = 1, 2 and 4 (curves 4–6), with heated MAZ-GST (*r* = 4, curve 2) and with BSA (*r* = 4, curve 3). It can be seen that neither heated MAZ-GST nor BSA change the melting profile of the quadruplex. In contrast, MAZ-GST promotes, in a dose-response manner, a complete extinction of the quadruplex-to-single strand transition. This strongly suggests that in the DNA–protein complex, the quadruplex structure is disrupted by MAZ-GST. When the GST moiety of the protein was incubated with the quadruplex, no effect on melting was observed (not shown).

We have also examined the impact of MAZ-GST on the parallel *hras*-2 quadruplex, at various KCl concentrations (10, 25, 50, 75, 100 and 140 mM) (Figure 4E). In this KCl concentration range, *hras*-2 is folded (spectra 1–6), and the distance between the fluorophores is ∼44 Å. The transformation of quadruplex *hras*-2 into a linear B-DNA conformation by its complementary strand (24Y) increases the fluorophore distance to ∼78 Å. In the presence of MAZ-GST (*r* = 4), the fluorescence of the donor increases (spectra 7–12), as the protein unfolds the quadruplex. In 10 mM KCl, the end-to-end distance jumps to ∼61 Å, which hints to a remarkable opening of the quadruplex in comparison with the distance between the two fluorophores in the corresponding *hras*-2 duplex (∼78 Å). At higher KCl concentrations the quadruplex stability increases and the unfolding becomes less efficient (the fluorophore distance decreases from 61 to 44 Å, inset). Figure 4F reports the FRET-melting experiments showing that quadruplex *hras*-2 has a *T*_M_ of 76, 83 or >90°C in 25, 50 and 100 mM KCl, respectively. When the samples before melting were treated for 2 h with MAZ-GST (*r* = 4), the melting curve at 25 mM KCl was totally abrogated, but at 50 and 100 mM KCl it was partly abrogated, as the quadruplex *T*_M_ is >80°C. In 100 mM NaCl, MAZ-GST unfolds quadruplex *hras*-2 more efficiently, as it has a lower *T*_M_ (60°C) (S_4_) and probably also a different topology.

As far as we know, our data demonstrate for the first time that MAZ, a zinc finger transcription factor recognizing blocks of guanine, is able to unfold G4-DNA. We tested the capacity of MAZ to unfold other well-known quadruplexes and found that the protein was quite active against the human telomere but not against the *CMYC* quadruplex (not shown).

Next we reasoned that the unfolding by MAZ-GST of the *HRAS* G-quadruplexes should facilitate their hybridization to the complementary pyrimidine strands (24 and 27Y) to yield the corresponding B-DNA duplexes. To explore if the quadruplex to B-DNA transformation at *hras*-1 and *hras*-2 is facilitated by MAZ-GST, we set up an electrophoretic assay (Figure 5). The hybridization of quadruplexes *hras*-1 and *hras*-2, end labeled at the 5′ end with ^32^P, to their complementary strands was followed in 100 and 30 mM KCl, respectively, because of their different stability. The quadruplexes incubated with two equivalents of complementary strand, are transformed into duplexes on a timescale of hours, at room temperature. In keeping with their different thermal stability, quadruplex *hras*-1 (*T*_M_ ∼53/65°C, in 100 mM KCl) hybridizes to complementary 27Y in about 2 h (lane 4, left gel), while quadruplex *hras*-2 (*T*_M_ ∼76°C, in 30 mM KCl) hybridizes to 24Y in a much longer time: after 8 h of incubation 60% *hras*-2 is still in quadruplex (lane 6, right gel). However, when the annealing reaction was carried out in the presence of excess recombinant MAZ-GST (*r* = 10), duplex formation was much faster. After an incubation of 0.1 h, quadruplex *hras*-1 was completely transformed into B-DNA (lane 8, left gel), while 23% of *hras*-2 was still in quadruplex (lane 8, right gel). The duplex formed during the annealing of quadruplex *hras*-2 to 24Y, bound to MAZ-GST, thanks to its high affinity for the protein, and formed the expected 1:1 and 1:2 complexes. In contrast, under the experimental conditions adopted, MAZ-GST did not stably bind to duplex *hras*-1, as this target contains a mismatched GGGCGGC MAZ-binding site of lower affinity (the smeared bands suggest a duplex dissociation from the protein). The percentage of formed *hras*-1 or *hras*-2 duplex (either free or bound to MAZ-GST) is reported as a function of time in Figure 5B. The plots show that in the presence of the complementary strands, an excess of MAZ-GST (*r* = 10) rapidly transforms both *hras*-1 and *hras*-2 quadruplexes into the respective B-DNA duplexes.

**Figure 6.**
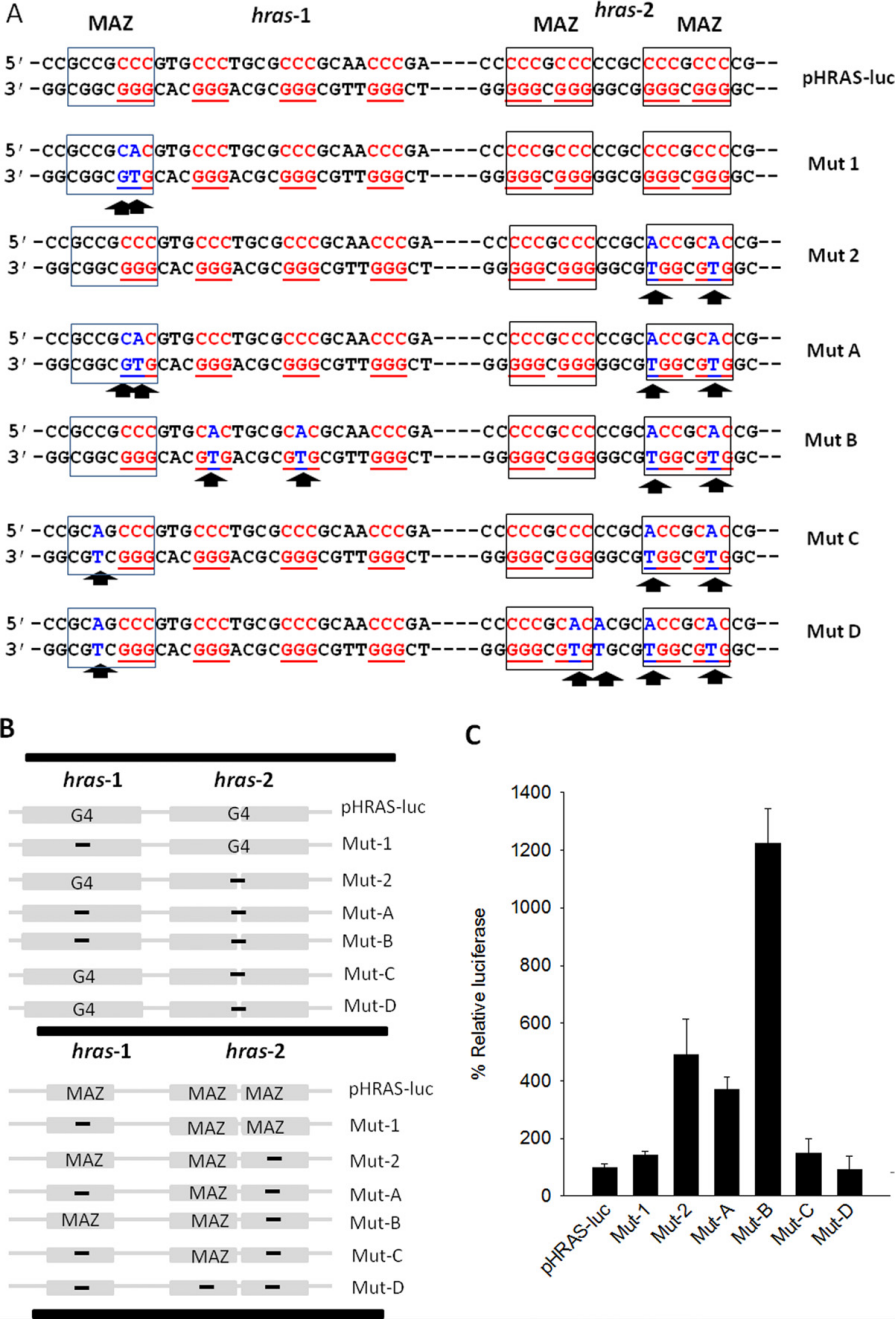
(**A**) Point mutations inserted in *hras*-1 and/or *hras*-2 of the designed mutant plasmids Mut-1, Mut-2, Mut-A, Mut-B, Mut-C and Mut-D. The sites of MAZ in *hras*-1 and *hras*-2 are signed with rectangles; (**B**) Scheme showing the presence of the quadruplex-forming motif (top panel) and MAZ-binding site (bottom panel) in the various plasmids. (**C**) % relative luciferase from wild-type and mutant plasmids.

To explore how the two neighboring G-quadruplexes, located upstream of TSS, affect *HRAS* transcription, we prepared a series of mutant plasmids, by introducing point mutations in the *hras*-1 and/or *hras*-2 G-elements (Figure 6A). We first constructed a plasmid, pHRAS-luc, bearing firefly luciferase driven by the human *HRAS* promoter (9). From pHRAS-luc we obtained by site-directed mutagenesis several mutant plasmids, where quadruplex formation was abolished at *hras*-1 (Mut-1) or *hras*-2 (Mut-2, Mut C, Mut D) or at both *hras*-1 and *hras*-2 (Mut-A and Mut-B). As the quadruplex-forming sequences overlap the MAZ-binding sites, these were also modified by the point mutations. The presence or absence in the constructed expression plasmids of G4-DNA and/or MAZ-binding sites at *hras*-1 and *hras*-2 is summarized in Figure 6B. Dual luciferase assays have been carried out by transfecting T24 bladder cancer cells with the constructed plasmids. The level of firefly luciferase expressed by the vectors was normalized to the level of *Renilla* luciferase and the data obtained were referred to the level of luciferase of wild type pHRAS-luc, which was set to 100 (Figure 6C). Analyzing the data in terms of quadruplex formation at *hras*-1 and *hras*-2, we could conclude that the two neighboring G-quadruplexes strongly lock the promoter in an inactive state. Indeed, when quadruplex formation is abolished at *hras*-1 or *hras*-2, luciferase increases to 143 (Mut-1) or 492 (Mut-2). This means that the parallel G-quadruplex structure at *hras*-2 is a stronger transcription repressor than the antiparallel G-quadruplex at *hras*-1. As we expected, there is a correlation between quadruplex stability and repressor activity. However, when both G-quadruplexes are abrogated, luciferase increases up to 1225 (Mut-B), indicating that the two G-quadruplexes extinct the promoter activity in a synergistic manner. It is interesting to observe that although Mut-A is not able to form either *hras*-1 or *hras*-2 quadruplex, its luciferase level increases only up to 370. We wondered why Mut-A and Mut-B express different levels of luciferase, though neither of them is capable to form quadruplexes at *hras*-1 and *hras*-2. The answer can be found in the scheme of Figure 6B, which shows the quadruplexes and MAZ-binding sites at *hras*-1 and *hras*-2, in the various expression plasmids. The G-elements in Mut-A and Mut-B can only assume a duplex conformation, thanks to the point mutations introduced (Figure 6A), but while Mut-B has maintained the MAZ-binding sites at both *hras*-1 and *hras*-2, Mut-A has lost the MAZ-binding site at *hras*-1. So, the data suggest that the simultaneous presence of the MAZ-binding sites at *hras*-1 and *hras*-2 is essential for optimal promoter activity and luciferase synthesis. When Mut-2, Mut-C and Mut-D are compared with each other, a similar conclusion can be drawn (Figure 6B). The three plasmids can only form quadruplex *hras*-1, but the two MAZ-binding sites in Mut-2 drop to one in Mut-C and to zero in Mut-D. The reduction of the number of MAZ-binding sites is accompanied by a luciferase decrease from 492 (Mut-2) to 149 (Mut-C) and 93 (Mut-D). Again, these data demonstrate that the activity of the *HRAS* promoter requires the presence of MAZ at both *hras*-1 and *hras*-2.

**Figure 7.**
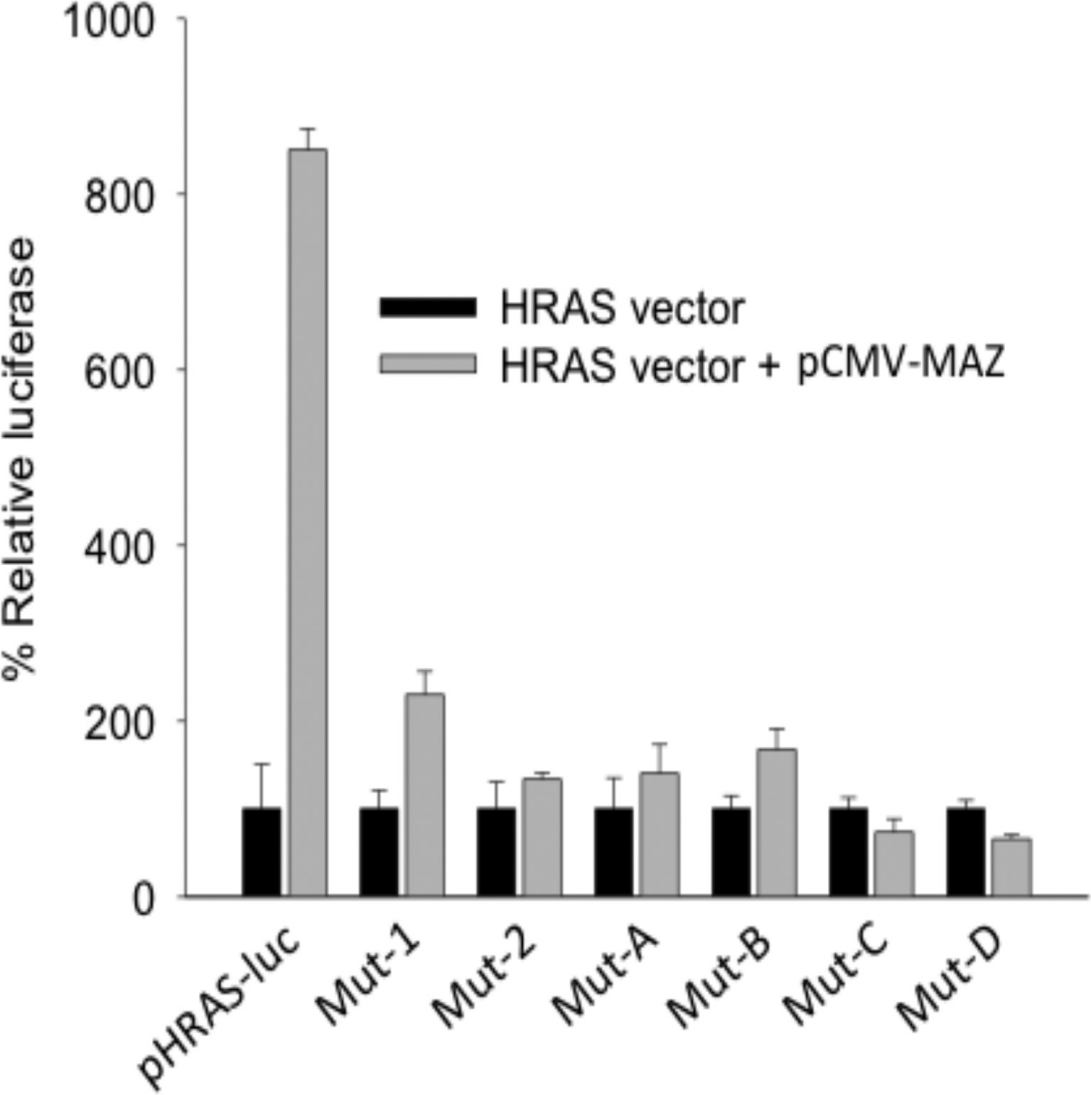
Dual luciferase assay in T24 cells transfected with wild-type pHRAS-luc, Mut-1, Mut-2, Mut-A, Mut-B, Mut-C and Mut-D (HRAS vector) or co-transfected with pCMV-MAZ (HRAS vector + pCMV-MAZ). The % relative luciferase reported in ordinate is given by: (L_HV_/L_HV+M_) x 100, where L_HV_ is the luciferase expressed by the HRAS vectors, L_HV+M_ is the luciferase expressed by the HRAS and pCMV-MAZ vectors.

We then asked how the wild-type and mutant *HRAS* promoters responded to the overexpression of MAZ. The wild type and mutant plasmids were co-transfected in T24 bladder cancer cells with pCMV-MAZ, a vector expressing MAZ. The relative increase of luciferase caused by MAZ is reported in Figure 7. It can be seen that wild-type pHRAS-luc strongly responded to the overexpression of MAZ, while Mut-1 gave a weak response and the other mutant plasmids practically did not respond. As stated above, in pHRAS-luc and Mut-1, the *HRAS* promoter is locked into an inactive state mainly by quadruplex *hras*-2, this structure being very stable under physiological conditions (*T*_M_ > 80°C). In the absence of any stimulus, the transcription rate of these vectors is quite low (see Figure 6C). But when MAZ is overexpressed, the protein promotes the transformation of the *hras*-1 and *hras*-2 quadruplexes into B-DNA. Consequently, the transcription block is removed and luciferase produced by pHRAS-luc increases 8-fold. Mut-1 responds to the MAZ overexpression less efficiently than the wild-type plasmid because the mutations delete the MAZ-binding site at *hras*-1 (optimal transcription requires both MAZ-binding sites at *hras*-1 and *hras*-2). By contrast, all the other mutant vectors, which do not have the block due to quadruplex *hras*-2 (see Figure 6B), are characterized by a high intrinsic transcription level. So, the overexpression of MAZ does not further increase transcription. The data confirm that *HRAS* transcription is strongly activated by MAZ, most likely through the unfolding of G4-DNA. We have also tried to overexpress MAZ and Sp1 together, obtaining roughly the same results as those observed with the overexpression of only MAZ (not shown).

**Figure 8.**
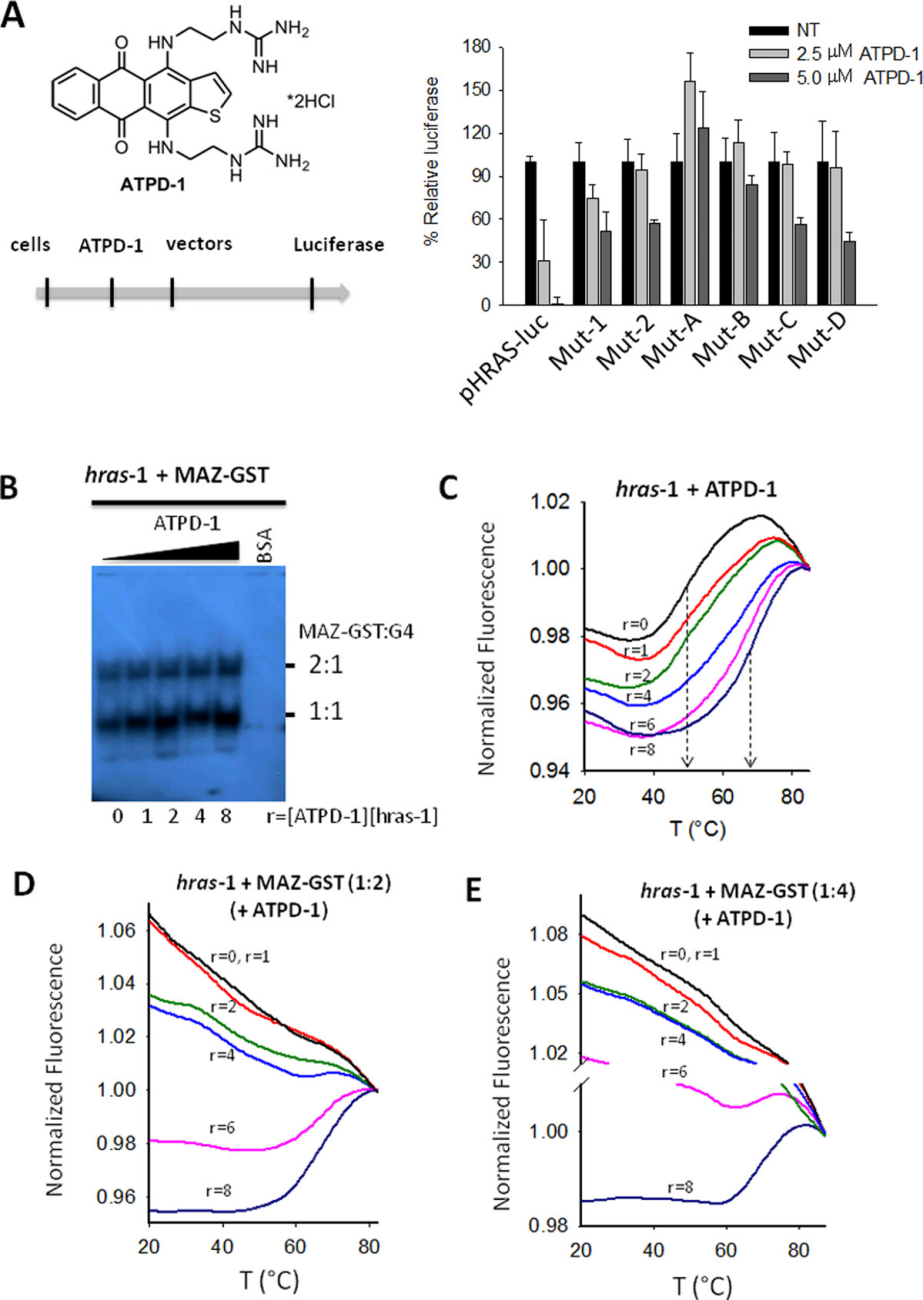
(**A**) Structure of ATPD-1 and scheme of the transfection experiment. T24 cells were treated with ATPD-1 for 24 h, then transfected with luciferase vector. Right panel shows the dual luciferase assay in T24 cells treated with increasing amounts of ATPD-1 (2.5 and 5 μM) and transfected with wild-type pHRAS-luc or mutant Mut-1, Mut-2, Mut-A, Mut-B, Mut-C and Mut-D; (**B**) EMSA showing the binding of MAZ-GST (2 μg) to 20 nM quadruplex *hras*-1 in binding buffer containing increasing amounts of ATPD-1 (*r* = 0, 1, 2, 4, 6 and 8); (**C**) FRET-melting of 200 nM *hras*-1 after overnight incubation in 50 mM KCl and ATPD-1 at *r* = 0, 1, 2, 4, 6, 8; (**D**) FRET-melting curves of *hras*-1 (200 nM) treated with ATPD-1 (*r* = 0, 1, 2, 4, 6, 8) and MAZ-GST (400 nM); (**E**) as in D, but with 800 nM MAZ-GST. The curves in (B), (D) and (E) have been normalized to the fluorescence at 80°C (or 85°C).

Given their critical role in transcription regulation, the two neighboring G-quadruplexes can be targeted by small ligands in order to downregulate *HRAS*: an oncogene playing a key role in the pathogenesis of urinary bladder cancer (5,6,33,34). These molecules should bind preferentially G4-DNA instead of B-DNA, and consequently repress transcription by increasing the stability of the G-quadruplexes. This should inhibit the unfolding of the quadruplex by MAZ and arrests cell growth. To support these hypotheses, we used an antrathiophenedione (ATPD-1), which was recently found to strongly bind to *hras*-1 (*K*_D_ of 0.34 ± 0.07, Δ*T*_M_ of 12 and 22°C, in 100 mM KCl) and *hras*-2 (*K*_D_ of 0.27 ± 0.02, Δ*T*_M_ of 19°C, in 10 mM KCl) quadruplexes (35). We first treated T24 cancer cells with ATPD-1 for 24 h, then with the expression vectors for further 48 h. At the end of the incubation a dual luciferase assay was carried out. The relative firefly luciferase values obtained with wild-type and mutant plasmids, in absence and presence of 2.5 and 5 μM ATPD-1, are reported in the histogram of Figure 8A. It shows that ATPD-1 completely represses, in a dose-response manner, luciferase of wild-type plasmid pHRAS-luc, bearing both *HRAS* quadruplex-forming motifs. In contrast, the ligand reduced by ∼50% the luciferase expressed by mutants Mut-1 and Mut-2, which bear only one quadruplex-forming motif. This suggests that only one G-quadruplex is unable to block transcription completely. As we expected, Mut-A and Mut-B being unable to form any G-quadruplex, do not respond to ATPD-1. Instead Mut-C and Mut-D respond to the ligand, as they have one quadruplex-forming motif (*hras*-1).

ATPD-1 can repress transcription either by competing with the binding of MAZ to the quadruplex structures or, alternatively, by enhancing the stability of the *HRAS* quadruplexes and thus making MAZ unable to unfold them. The first hypothesis was tested by an electrophoretic mobility shift assay. Figure 8B shows that quadruplex *hras*-1 and MAZ-GST form two complexes with 1:1 and 1:2 stoichiometries. The formation of these complexes is not inhibited by ATPD-1 at *r* = 0, 1, 2, 4, 6 and 8 (*r* = [ATPD-1]/[hras-1]). This suggests that the ligand does not compete with MAZ-GST for binding to the quadruplexes. A similar result was obtained with quadruplex *hras*-2 (S_5_). We therefore focused on the second hypothesis and tested if MAZ-GST is able to unfold the *HRAS* G-quadruplexes in the presence of ATPD-1. Figure 8C shows that an excess of ATPD-1 over the *hras*-1 quadruplex (up to 8-fold), increases the quadruplex *T*_M_ from ∼51/65 to 70°C, in 50 mM KCl. When the G-quadruplex was treated with either 2 or 4 equivalents of MAZ-GST in the presence of increasing amounts of ATPD-1 (*r* = 0, 1, 2, 4, 6 and 8), it was completely disrupted at *r* = 1, 2 and 4, and partially disrupted at *r* = 6. In contrast, when ATPD-1 was used at *r* = 8, MAZ-GST was unable to unfold the G-quadruplex (Figure 8D, E). This experiment provides a mechanistic insight into the capacity of ATPD-1 to lower *HRAS* expression and arrest cell growth in bladder cancer (35).

Our findings suggest that the two G-elements controlling the expression of *HRAS* may serve as effective targets for antigene G-quadruplex ligands.

## CONCLUSION

In this study we have reported a systematic mutational analysis of the *HRAS* promoter at *hras*-1 and *hras*-2, by dissecting the quadruplex-forming motifs from the MAZ-binding sites. The results allow us to conclude that two neighboring G-quadruplexes upstream of TSS repress transcription in a coordinated and efficient way. For the first time we demonstrate by FRET experiments that recombinant MAZ unfolds the *HRAS* G-quadruplexes. Thanks to this ability, MAZ facilitates the hybridization of the G-quadruplexes to the complementary strand, in order to reconstitute the B-DNA duplex. Our data suggest that MAZ, a zinc-finger transcription factor that recognizes runs of guanines, has two functions in the *HRAS* promoter: remove the transcription blockade caused by the neighboring G-quadruplexes at *hras*-1 and *hras*-2, and activate transcription. In agreement with the model proposed for *CMYC* (36), the duplex-to-quadruplex transformation is likely to behave as an on–off switch that controls transcription. This switch is an attractive target for small therapeutic molecules. We have indeed observed a dramatic arrest of *HRAS* transcription when we used a cell-penetrating anthrathiophenedione G4-DNA ligand (ATPD-1) which tightly binds to the *HRAS*-quadruplexes. The two neighboring G-quadruplexes that control transcription promise to be attractive targets for anticancer drugs specific for oncogenic *HRAS*: the key genetic lesion leading to urinary bladder cancer in human.

## SUPPLEMENTARY DATA

Supplementary Data are available at NAR Online.

SUPPLEMENTARY DATA

## References

[1] Lowy D.R., Willumsen B.M. (1993). Function and regulation of ras. Annu. Rev. Biochem..

[2] Downward J. (1998). Ras signalling and apoptosis. Curr. Opin. Genet. Dev..

[3] Porter A.C., Vaillancourt R.R. (1998). Tyrosine kinase receptor-activated signal transduction pathways which lead to oncogenesis. Oncogene.

[4] Schubbert S., Shannon K., Bollag G. (2007). Hyperactive Ras in developmental disorders and cancer. Nat. Rev. Cancer.

[5] Vageli D., Kiaris H., Delakas D., Anezinis P., Cranidis A., Spandidos D.A. (1996). Transcriptional activation of H-ras, K-ras and N-ras proto-oncogenes in human bladder tumors. Cancer Lett..

[6] Theodorescu D., Cornil I., Fernandez B.J., Kerbel R. (1990). Overexpression of normal and mutated forms of HRAS induces orthotopic bladder invasion in a human transitional cell carcinoma. Proc. Natl. Acad. Sci. U.S.A..

[7] Mo L., Zheng X., Huang H.Y., Shapiro E., Lepor H., Cordon-Cardo C., Sun T.T., Wu X.R. (2007). Hyperactivation of Ha-ras oncogene, but not Ink4a/Arf deficiency, triggers bladder tumorigenesis. J. Clin. Invest..

[8] Baines A.T., Depeng X., Der C.J. (2011). Inhibition of Ras for cancer treatment: the search continue. Future Med. Chem..

[9] Membrino A., Cogoi S., Pedersen E.B., Xodo L.E. (2011). G4-DNA formation in the HRAS promoter and rational design of decoy oligonucleotides for cancer therapy. PLoS One.

[10] Todd A.K., Neidle S. (2008). The relationship of potential G-quadruplex sequences in cis-upstream regions of the human genome to SP1-binding elements. Nucleic Acids Res..

[11] Kumar P., Yadav V.K., Baral A., Kumar P., Saha D., Chowdhury S. (2011). Zinc-finger transcription factors are associated with guanine quadruplex motifs in human, chimpanzee, mouse and rat promoters genome-wide. Nucleic Acids Res..

[12] Cogoi S., Paramasivam M., Membrino A., Yokoyama K.K., Xodo L.E. (2010). The KRAS promoter responds to Myc-associated zinc finger and poly(ADP-ribose) polymerase 1 proteins, which recognize a critical quadruplex-forming GA-element. J. Biol. Chem..

[13] Cogoi S., Zorzet S., Rapozzi V., Géci I., Pedersen E.B., Xodo L.E. (2013). MAZ-binding G4-decoy with locked nucleic acid and twisted intercalating nucleic acid modifications suppresses KRAS in pancreatic cancer cells and delays tumor growth in mice. Nucleic Acids Res..

[14] Leroy C., Manen D., Rizzoli R., Lombès M., Silve C. (2004). Functional importance of Myc-associated zinc finger protein for the human parathyroid hormone (PTH)/PTH-related peptide receptor-1 P2 promoter constitutive activity. J. Mol. Endocrinol..

[15] Song H., Claycomb R., Tai T.C., Wong D.L. (2003). Regulation of the Rat phenylethanolamine N-methyltransferase gene by transcription factors Sp1 and MAZ. Mol. Pharmacol..

[16] Himeda C.L., Ranish J.A., Hauschka S.D. (2008). Quantitative proteomic identification of MAZ as a transcriptional regulator of muscle-specific genes in skeletal and cardiac myocytes. Mol. Cell. Biol..

[17] Palumbo S.L., Memmott R.M., Uribe D.J., Krotova-Khan Y., Hurley L.H., Ebbinghaus S.W. (2008). A novel G-quadruplex-forming GGA repeat region in the c-myb promoter is a critical regulator of promoter activity. Nucleic Acids Res..

[18] Chapados B.R., Hosfield D.J., Han S., Qiu J., Yelent B., Shen B., Tainer J.A. (2004). Structural basis for FEN-1 substrate specificity and PCNA-mediated activation in DNA replication and repair. Cell.

[19] Sohon S.Y., Bae W.J., kim J.J., Yeom K-H, kim V.N., Cho Y. (2007). Crystal structure of human DGCR8 core. Nat. Struct. Mol. Biol..

[20] Mergny J.L., Maurizot J.C. (2001). Fluorescence resonance energy transfer as a probe for G-quartet formation by a telomeric repeat. Chembiochem.

[21] Wong I., Lohman T.M. (1993). A double-filter method for nitrocellulose-filter binding: application to protein-nucleic acid interactions. Proc. Natl. Acad. Sci. U.S.A..

[22] Ishii S., Merlino G.T., Pastan I. (1985). Promoter region of the human Harvey ras proto-oncogene: similarity to the EGF receptor proto-oncogene promoter. Science.

[23] Ishii S., Kadonaga J.T., Tjian R., Brady J.N., Merlino G.T., Pastan I. (1986). Binding of the Sp1 transcription factor by the human Harvey ras1 proto-oncogene promoter. Science.

[24] Parks C.L., Shenk T. (1996). The serotonin 1a receptor gene contains a TATA-less promoter that responds to MAZ and Sp1. J. Biol. Chem..

[25] Baral A., Kumar P., Halder R., Mani P., Yadav V.K., Singh A., Das S.K., Chowdhury S. (2012). Quadruplex-single nucleotide polymorphisms (Quad-SNP) influence gene expression difference among individuals. Nucleic Acids Res..

[26] Paramasivam M., Membrino A., Cogoi S., Fukuda H., Nakagama H., Xodo L.E. (2009). Protein hnRNP A1 and its derivative Up1 unfold quadruplex DNA in the human KRAS promoter: implications for transcription. Nucleic Acids Res..

[27] Federici L., Arcovito A., Scaglione G.L., Scaloni F., Lo Sterzo C., Di Matteo A., Falini B., Giardina B., Brunori M. (2010). 1.Nucleophosmin C-terminal leukemia-associated domain interacts with G-rich quadruplex forming DNA. J. Biol. Chem..

[28] González V., Guo K., Hurley L., Sun D. (2009). Identification and characterization of nucleolin as a c-myc G-quadruplex-binding protein. J. Biol. Chem..

[29] Raiber E.A., Kranaster R., Lam E., Nikan M., Balasubramanian S. (2012). A non-canonical DNA structure is a binding motif for the transcription factor SP1 in vitro. Nucleic Acids Res..

[30] Salas T.R., Petruseva I., Lavrik O., Bourdoncle A., Mergny J.L., Favre A., Saintomé C. (2006). Human replication protein A unfolds telomeric G-quadruplexes. Nucleic Acids Res..

[31] Seidel C.A.M., Schulz A., Sauer M.M.H. (1996). Nucleobase-specific quenching of fluorescent dye. 1. Nucleobase one-electron redox potentials and their correlation with static and dynamic efficiencies. J. Phys. Chem..

[32] Collie G.W., Parkinson G.N. (2011). The application of DNA and RNA quadruplexes to therapeutic medicines. Chem. Soc. Rev..

[33] Choudhary S., Wang K.K., Wang H.C. (2011). Oncogenic H-Ras, FK228, and exogenous H2O2 cooperatively activated the ERK pathway in selective induction of human urinary bladder cancer J82 cell death. Mol. Carcinog..

[34] Boulalas I., Zaravinos A., Karyotis I., Delakas D., Spandidos D.A. (2009). Activation of RAS family genes in urothelial carcinoma. J. Urol..

[35] Cogoi S., Shchekotikhin A.E., Membrino M., Sinkevich Y.B., Xodo L.E. (2013). Guanidino anthrathiophenediones as G-quadruplex binders: uptake, intracellular localization, and anti-Harvey-Ras gene activity in bladder cancer cells. J. Med. Chem..

[36] Brooks T.A., Hurley L.H. (2009). The role of supercoiling in transcriptional control of MYC and its importance in molecular therapeutics. Nat. Rev..

