# Supplementary material for: HRAS is silenced by two neighboring G-quadruplexes and activated by MAZ, a zinc-finger transcription factor with DNA unfolding property
Source: Nucleic Acids Res. 2014 Jul 9;42(13):8379–88. doi: 10.1093/nar/gku574 (PMC4117790; doi:10.1093/nar/gku574)
Supplement: SUPPLEMENTARY DATA [file supp_42_13_8379__index.html]

 HRAS is silenced by two neighboring G-quadruplexes and activated by MAZ, a zinc-finger transcription factor with DNA unfolding property — SUPPLEMENTARY DATA 

# *HRAS* is silenced by two neighboring G-quadruplexes and activated by MAZ, a zinc-finger transcription factor with DNA unfolding property

## SUPPLEMENTARY DATA

**Files in this Data Supplement:**

- Supplementary Data
