# Supplementary material for: HRAS is silenced by two neighboring G-quadruplexes and activated by MAZ, a zinc-finger transcription factor with DNA unfolding property
Source: Nucleic Acids Res. 2014 Jul 9;42(13):8379–88. doi: 10.1093/nar/gku574 (PMC4117790; doi:10.1093/nar/gku574)
Supplement: SUPPLEMENTARY DATA [file supp_gku574_nar-00220-f-2014-File002.docx]

**SUPPORTING INFORMATION**

***HRAS* is silenced by two neighboring G-quadruplexes and activated by MAZ, a transcription factor with DNA unfolding property**

Susanna Cogoi ^1^, Andrey E. Shchekotikhin^2^ and Luigi E. Xodo^1^*

^1^Department of Medical and Biological Sciences, School of Medicine, P.le Kolbe 4, 33100 Udine, Italy;

^2^Gause Institute of New Antibiotics, Russian Academy of Medical Sciences, B. Pirogovskaya, 11, Moscow 119021, Russia;

***Corresponding Author**

Luigi E Xodo, Department of Medical and Biological Sciences, P.le Kolbe 4, University of Udine, Udine 33100, Italy, E-mail: [luigi.xodo@uniud.it](mailto:luigi.xodo@uniud.it); Tel +39.0432.494395, Fax: +39.0432.494301;

**Supporting Information S_1_**


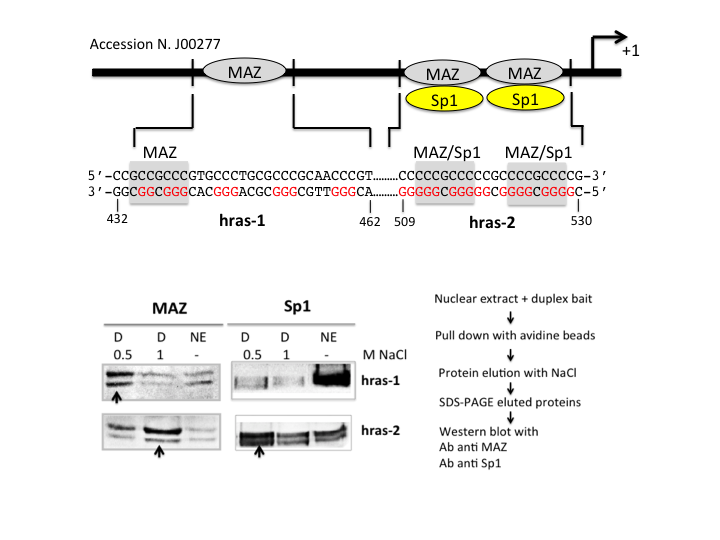


**Pull-down and Western blot assay with T24 nuclear extract and duplex *hras*-1 or duplex *hras*-2 as molecular baits for MAZ and Sp1.**

A 36-mer biotinylated oligonucleotide containing either the sequence of *hras*-1 or *hras*-2, was hybridized to its complementary strand (bait) and incubated with a nuclear extract of T24 bladder cancer cells, which harbor mutant *HRAS*. The DNA-protein complexes formed between the bait and the extract were pulled down with streptavidin-functionalized paramagnetic beads (Promega, Milan, Italy). The proteins were then eluted from the beads with 0.5 and 1 M NaCl solutions, separated in 10% SDS-PAGE and blotted overnight in 25 mM Tris, 192 mM Glycine and 20% methanol at 4°C on a nitrocellulose membrane. The membrane was incubated with anti-MAZ H-50 or anti-Sp1 H-225 antibodies diluted 1:200 (Santa Cruz). Secondary antibody used was anti-rabbit IgG Peroxidase conjugate, diluted 1: 10000 (Calbiochem). The antibodies were diluted in 10 mM Tris pH 7.9, 150 mM NaCl, 0.05% Tween (TBST). The signal was developed with Super Signal®West PICO, and Super Signal®West FEMTO (Pierce) and detected with ChemiDOC XRS. The protein levels were quantified by Quantity ONE 4.6.5 software (Bio-Rad Laboratories, CA, USA).

The results obtained, reported in Figure S1, show that the fractions eluted from duplex hras-2 were enriched with MAZ and Sp1, while those eluted from duplex hras-1 were enriched of only MAZ, suggesting that Sp1 does not seem to have affinity for the site. In agreement with the ChIP data previously obtained (ref 9, text), the pull-down assay indicated that duplex *hras*-2 is bound by Sp1 and MAZ, while duplex hras-1 only by MAZ. Moreover, the fact that MAZ is eluted from duplex *hras*-2 at 1 M NaCl, while it is eluted from duplex hras-1 at 0.5 M, suggests that MAZ has more affinity for the former duplex than the later one. Indeed, in duplex hras-1 the MAZ-binding site contains one mismatch: 5’-GGGCGGC , while duplex hras-2 contains two perfect MAZ-binding site 5’-GGGCGGG.

**Supporting Information, S_2a_**


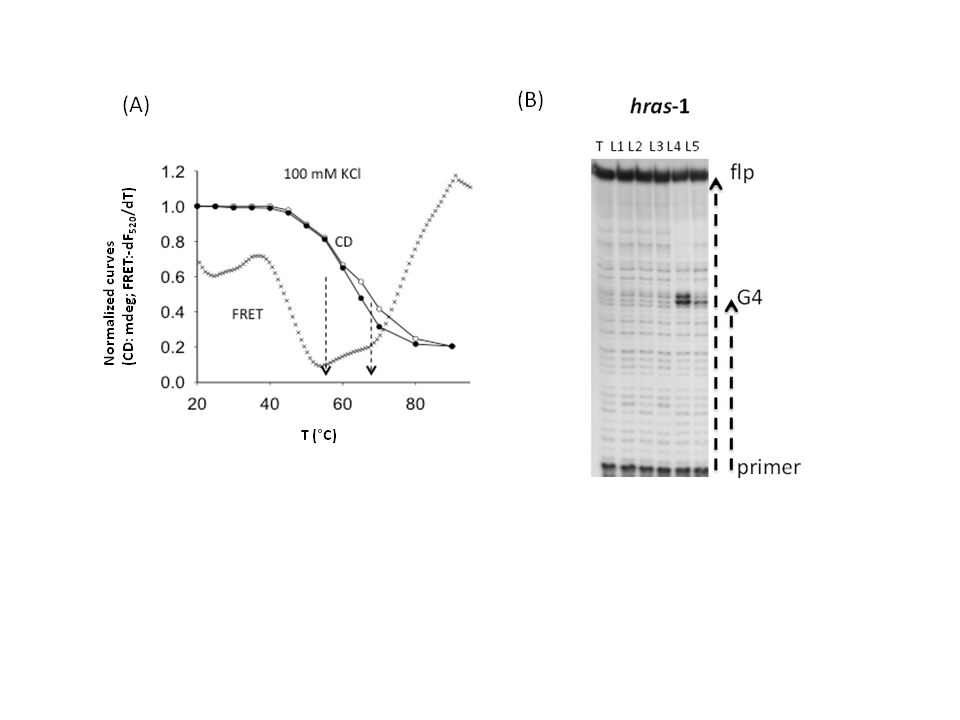


(C)


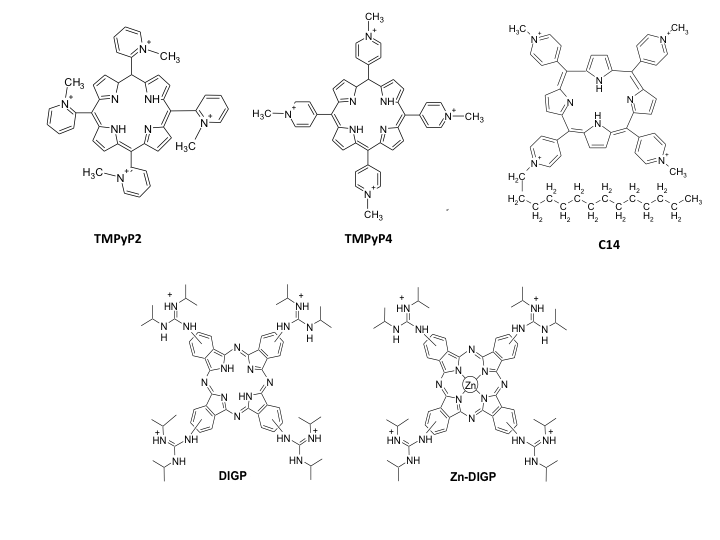


1. **Melting curves of hras-1 in 50 mM Tris-HCl pH 7.4, 100 mM KCl.**

The graph shows heating/cooling curves of hras-1 obtained from CD spectra as a function of temperature. The 290 nm ellipticity of 3 μM hras-1 was plotted against temperature (20, 25, 30, 35, 40, 45, 50, 55, 60, 70, 80, 90 °C). Note that the heating curve is biphasic, while the cooling curve is not. The cooling curve was obtained from spectra recorded soon after the temperature reached the new value. The spectra have been normalized to the ellipticity at 20°C. The same graph reports the heating FRET-melting curve of 200 nM hras-1 labeled with FAM and TAMRA at the 5’ and 3’ ends, in 100 mM KCl, 50 mM Tris-HCl. The FRET-melting curve is biphasic with *T*_M_’s at ~53 and ~65 °C. DMS-footprinting and CD suggest that hras-1 could form LLL and LLD antiparallel G-quadruplex structures;

1. **Primer extension assay with 82-mer template containing the hras-1 motif**

A single-stranded DNA template of 82 nucleotides, containing the G-element *hras*-1, was used as template for Taq polymerase primer-extension reactions. The template has been purified by PAGE under denaturing conditions. The template (25 nM) was mixed with the ^32^P-labelled primer (25 nM), in the presence or absence of ligands (L1=TMPyP4, L2=TMPyP2, L3=C14, L4=Pc, L5=Zn-Pc), in 25 mM KCl, Taq buffer 1X and incubated overnight at 37°C. The primer extension reactions have been carried out for 1h, by adding 10 mM DTT, 100 mM dATP, dGTP, dTTP, dCTP and 3.75U of Taq polymerase (Euro Taq, Euroclone, Milan). The reactions were stopped by adding an equal volume of stop buffer (95% formamide, 10mM EDTA, 10mM NaOH, 0.1% xylene cyanol, 0.1% bromophenol blue). The products were separated on a 12% polyacrylamide sequencing gel prepared in TBE1X, 8 M urea. The gel was dried and exposed to autoradiography. Standard dideoxy sequencing reactions were performed to detect the exact positions in which DNA polymerase was arrested.

1. **Structure of the ligands used in the primer extension experiments**

**Supporting Information S_2,b_**

**
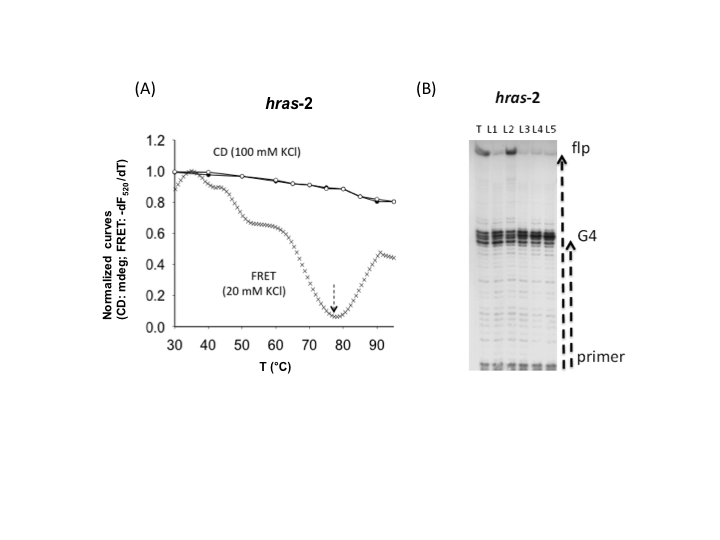
**

1. **Melting curves of hras-2 in 50 mM Tris-HCl pH 7.4, 20 and 100 mM KCl.**

The graph shows heating/cooling curves of hras-2 obtained from CD spectra as a function of temperature. The 290 nm ellipticity of 3 μM hras-2 was plotted against temperature (30, 40, 50, 60, 65, 70, 75, 80, 85, 90, 95 °C). Note that the heating and cooling curves are superimposable. Both indicate that in 100 mM KCl the hras-2 quadruplex is so stable that it does not melt. The same graph reports the heating FRET-melting curve of 200 nM hras-1 labeled with FAM and TAMRA at the 5’ and 3’ ends, in 20 mM KCl, 50 mM Tris-HCl. The FRET-melting curve of ~77 °C. DMS-footprinting and CD suggest that hras-1 could form a parallel G-quadruplex structure with 1/4/1 loops;

(B) **Primer extension assay with 78-mer template containing the hras-2 motif.** Experiment carried out as described above.

**Suporting Information S_3_**

**
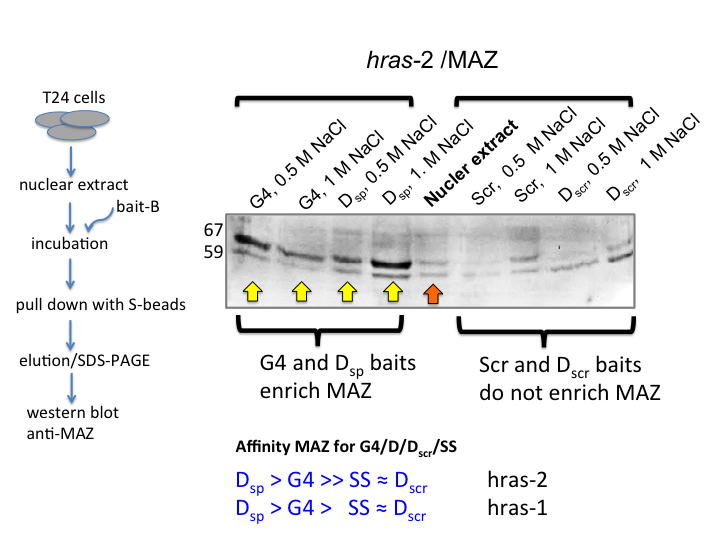
**

**Pull-down and Western blot assay with T24 nuclear extract and DNA probe hras-2 in duplex and quadruplex conformation.**

The experiment was performed using the protocol reported in S_1_ and following the scheme reported in the left of the figure S_5_. The results show that the fractions eluted from the G-quadruplex and duplex hras-2 probes (G4 and Dsp) are enriched of MAZ, while the fractions eluted from scramble sequence probes (SS and Dscr) are not. Moreover, the fact that MAZ is eluted from duplex hras-2 (Dsp) at 1 M NaCl, while it is eluted from G4 at 0.5 M, suggests that it has more affinity for duplex hras-2 than quadruplex hras-2. Note that MAZ exhibits a sequence specific binding to hras-2, as it shows no affinity for scramble sequences both in duplex or single-stranded forms.

**Supporting information S_4_**

**
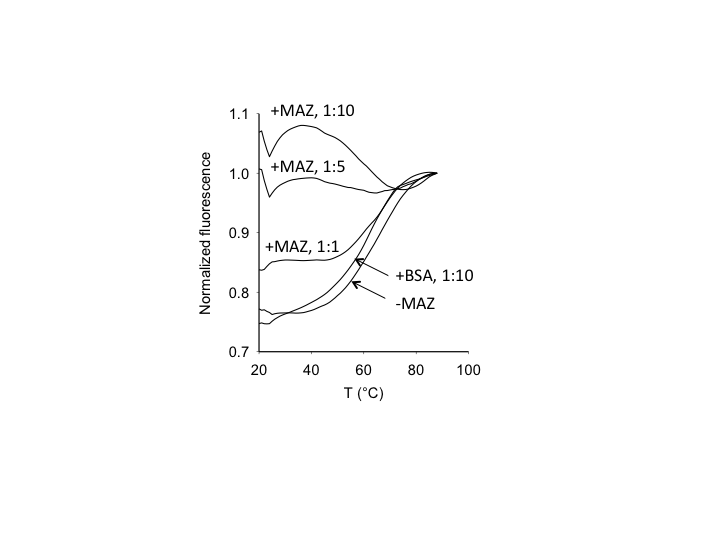
**

**FRET-melting curves of quadruplex hras-2 in 50 mM Tris-HCl pH 7.4, 100 mM NaCl, in the presence and absence of MAZ-GST.**

Quadruplex hras-2 (200 nM) was mixed in the above buffer with MAZ-GST at protein/DNA ratios of 1:1; 1:5 and 1:10. The samples treated with the protein were incubated for 2h at 37 °C before recording the melting curves. The quadruplex was also mixed with BSA at 1:10 ratio. MAZ-GST strongly modifies the melting curves. A 5-fold excess protein abrogates the melting curve, as one expect when the quadruplex is unfolded.

**Supporting information S_5_**

**_
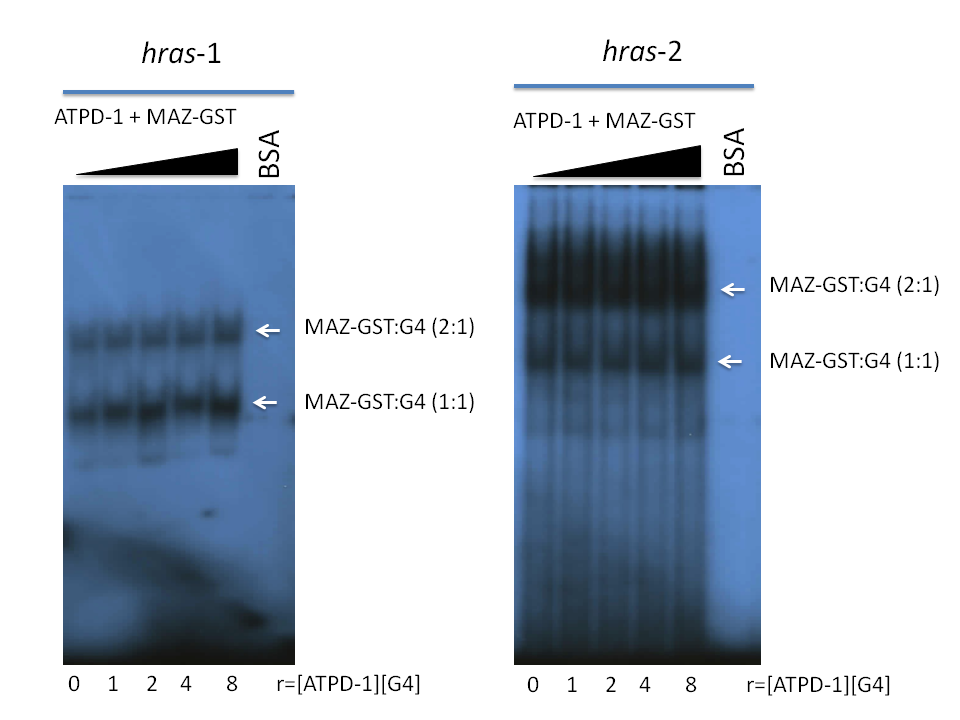
_**

Electrophoretic mobility shift assay (EMSA) of mixtures containing ^32^P-labelled quadruplex *hras*-2 (20 nM), MAZ-GST (2 μg) and ATPD-1 at r=0, 1, 2, 4 and 8 (r= [ATPD-1]/[quadruplex]). The reactions have been prepared in binding buffer (see Materials and Methods). Electrophoresis was carried out in TBE, 8% PAGE and 20 °C.

Quadruplex *hras*-2 and MAZ-GST form two complexes with 1:1 and 1:2 stoichiometries. Although ATPD-1 binds to the quadruplex, it does not compete with MAZ-GST for binding to the quadruplex.
